# How to design and implement a university-based COVID-19 testing programme? An evaluation of a novel RT-LAMP COVID-19 testing programme in a UK university

**DOI:** 10.1186/s12913-022-08717-5

**Published:** 2022-12-09

**Authors:** Claire Blackmore, Gareth W. Hall, Rebecca C. Allsopp, Anna L. Hansell, Caroline M. Cowley, Ruth C. Barber, Christopher W. Holmes, Martin D. Tobin, Jacqui A. Shaw, Nigel J. Brunskill, Philip N. Baker

**Affiliations:** 1 Centre for Environmental Health and Sustainability, University of Leicester, University Road, Leicester, LE1 7RH UK; 2 College of Life Sciences, University of Leicester, Leicester, UK; 3 Department of Genetics and Genome Biology, Leicester Cancer Research Centre, University of Leicester, Leicester, UK; 4 NIHR Health Protection Research Unit (HPRU) in Environmental Exposures and Health, University of Leicester, Leicester, UK; 5 Leicester Molecular Diagnostics, Leicester Cancer Research Centre, University of Leicester, Leicester, UK; 6 Leicester Precision Medicine Institute, University of Leicester, Leicester, UK; 7 University Hospitals of Leicester NHS Trust, Leicester, UK; 8 Department of Respiratory Sciences, University of Leicester, Leicester, UK; 9 Genetic Epidemiology and Public Health, Department of Health Sciences, University of Leicester, Leicester, UK; 10 Leicester NIHR Biomedical Research Centre, Leicester, UK; 11 Department of Genetics and Genome Biology, Leicester Cancer Research Centre, Translational Cancer Genetics, University of Leicester, Leicester, UK; 12 Department of Cardiovascular Sciences, University of Leicester, Leicester, UK; 13 Research & Enterprise, Fielding Johnson Building, University of Leicester, Leicester, UK

**Keywords:** COVID-19, University, Education, Mass testing, LAMP testing

## Abstract

**Background:**

Little is known about how asymptomatic testing as a method to control transmission of COVID-19 can be implemented, and the prevalence of asymptomatic infection within university populations. The objective of this study was to investigate how to effectively set-up and implement a COVID-19 testing programme using novel reverse transcriptase loop-mediated isothermal amplification (RT-LAMP) technology and to quantify the scale of asymptomatic infection on a university campus.

**Methods:**

An observational study to describe the set-up and implementation of a novel COVID-19 testing programme on a UK university campus between September and December 2020. RT-LAMP testing was used to identify asymptomatic cases.

**Results:**

A total of 1,673 tests were performed using RT-LAMP during the study period, of which 9 were positive for COVID-19, giving an overall positivity rate of 0.54%, equivalent to a rate in the tested population of 538 cases per 100,000 over the duration of testing. All positive tests were found to be positive on RT-PCR testing, giving a false positive rate of 0%.

**Conclusions:**

This study shows that it is possible to rapidly setup a universal university testing programme for COVID-19 in collaboration with local healthcare providers using RT-LAMP testing. Positive results were comparable to those in the local population, though with a different peak of infection. Further research to inform the design of the testing programme includes focus groups of those who underwent testing and further interrogation of the demographics of those opting to be tested to identify potential access problems or inequalities.

**Supplementary Information:**

The online version contains supplementary material available at 10.1186/s12913-022-08717-5.

## Background

First reported in December 2019, COVID-19 spread rapidly around the globe. It has caused widespread disruption, with countries implementing different measures to control the virus and limit its impact on healthcare and economies. There has often been a balancing act between keeping facilities and amenities open and controlling the transmission of the virus [1]. This has been particularly debated in the context of students studying in universities [2], where there have been calls for students to avoid universities [3] and the UK Independent SAGE Group (a group providing independent scientific advice to the UK government and public on COVID-19) advocated testing of university staff and students on arrival at university to identify pre-symptomatic or asymptomatic cases [4].

### Asymptomatic transmission

Although studies have found 17–20% of COVID-19 infections are asymptomatic across all population groups [5], this proportion rises significantly in younger and healthier groups [6]. Research into other coronaviruses, namely SARS and MERS, suggested that presymptomatic transmission was not a significant contributor to infection rates [7]. However, with COVID-19 infections, it has been shown that cases are also able to transmit the virus before they develop symptoms [8]. This has been particularly concerning as initial methods to control the virus had relied on isolation of people who were displaying symptoms. Estimates have suggested that up to 30% of COVID-19 infections could stem from asymptomatic transmission [9], leading to a focus on mass testing in asymptomatic populations in order to isolate those cases to break the chain of transmission. At the time of this study, national policy in England was that only people displaying symptoms, or those who had been advised by a healthcare professional, were eligible for COVID-19 testing using RT-PCR tests [10]. Rapid antigen tests were not widespread, with use trialled from August 2020 and limited to care home workers and hospital workers [11].

### University transmission

The return of university students to campuses in both Europe and America coincided with a spike in transmission in many countries. Correlations were seen between rises in COVID-19 cases in counties in America with increasing numbers of students within the local population [12]. Whilst the university population tend to be in younger age groups where the fatality rate from COVID-19 is much lower [13], there is often mixing with the local population: the CON-QUEST survey at University of Bristol found that around 40% of student contacts were with individuals not affiliated with the university [14]. This suggests that outbreaks in students can easily spread to older adults and other higher risk groups in the wider community.

### University testing programmes

A number of mitigation measures have been suggested and modelled [15], with some universities in the UK implementing their own asymptomatic testing programmes to reduce COVID-19 transmission on campus, although not recommended by the UK government at the time. Examples of this include the University of Cambridge who used pooled weekly RT-PCR tests in their “Stay Safe Cambridge Uni” programme [16], and the University of Nottingham who had a weekly testing programme in addition to a programme of rolling sentinel surveillance testing sessions in their “Test to Protect” scheme [17]. The testing programme at the University of East Anglia was credited with nearly eliminating COVID-19 on campus [18], and University of Southampton implemented novel reverse transcriptase loop-mediated isothermal amplification (RT-LAMP) testing on saliva samples to test asymptomatic staff and students [19].

**Fig. 1:**
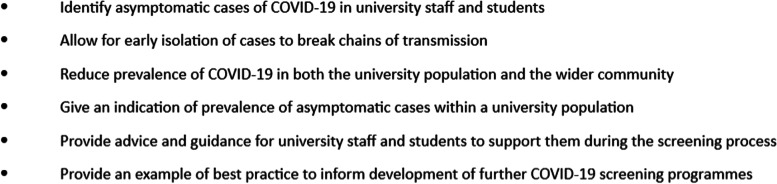
Aims and objectives of the university testing programme for COVID-19

This article details the design and set-up of a university testing programme to identify asymptomatic cases of COVID-19 within the staff and student population of the University of Leicester and to isolate and perform contact tracing within a timely manner. The rationale of this was to reduce transmission of COVID-19 and prevent outbreaks both in the university population and the wider community, enabling campus to remain open throughout the semester. Aims and objectives of the programme are detailed in Fig. 1.

## Methods

### Programme set up

The asymptomatic testing programme at the University of Leicester was designed and set up between July and September 2020, with the first participants being screened in September 2020. A steering group from within the University were able to capitalise on strong links between the University, the local hospital (University Hospitals of Leicester NHS Trust), and the local public health teams (Leicester City Council and Leicestershire County Council). Due to the rapid timeframe involved there was a need to simplify processes and use existing systems and it was not possible to trial the process with a pilot programme prior to the return of students to campus. Predicted uptake was unknown but preparations were made for up to 80% of staff and students to participate in testing. No restrictions were placed on frequency of testing. The testing programme received attention in local media and was included in communications to students enrolling at the university. To continuing students and staff, they were informed of the testing programme by email and details were also included in the university webpages providing information on COVID-19.

### LAMP testing, technique used, specificity and sensitivity

The testing programme set up at the University of Leicester used reverse transcriptase loop-mediated isothermal amplification (RT-LAMP). Contrary to RT-PCR, LAMP amplification [20] is performed at a single temperature on a basic thermocycler and uses reagents distinct from RT-PCR. Following increased demand and delays associated with centralised RT-PCR testing during the COVID-19 pandemic, it has been suggested that alternative testing modalities are required in the pandemic response [21]. An additional strength of RT-LAMP testing is that RT-LAMP assays use a longer region of the target DNA/RNA than real-time RT-PCR assays so the probability of detecting a fragmented target is lower, and it has been found to give results within minutes [22]. RT-LAMP is therefore a valuable diagnostic tool for SARS-CoV-2.

Swabs of the oropharyngeal and nasopharyngeal cavities were taken using Miraclean swabs placed into PrimeStore Molecular Transport Medium for viral inactivation and RNA stabilisation at room temperature [23]. Within a 24-h period, total nucleic acid extraction was followed by RT-LAMP against a single target. Total nucleic acid extraction to RT-LAMP was performed in-house using medium throughput automation and performed following quality standards, with guidance provided by Leicester Molecular Diagnostic Laboratory. The RT-LAMP assay implemented [24] was validated in-house using residual RNA from University Hospital Leicester (UHL) NHS inpatient swab samples with corresponding RT-PCR Ct value [25]. Furthermore, screening was performed to ISO 15189:2012 standards, using NHS IT infrastructure. Insufficient sample collection or sample extraction were identified as a major potential source of false negative results prior to testing commencing. To mitigate this, an internal control (total RNA) was used for each sample.

### Set up of testing centre and transport to laboratory

Due to safety concerns relating to the presence of guanidine thiocyanate in the molecular transport media, testing was carried out by participants in a supervised testing centre rather than participants being provided with testing kits. A testing centre was set up in a repurposed area of a building on the university campus. Eight individual booths were constructed to allow for simultaneous sample collection (see Supplementary Materials). Two sessions were run per day, each of three hours’ duration, with ten minutes allowed per appointment. This was adequate for sample collection and cleaning of the booth. Samples were transported to the laboratory at the end of each testing session. Sample batches arrived at the laboratory for same day RNA extraction and RT-LAMP. Data analysis and results notification followed (same day or next day depending on time of sample batch delivery): a turn-around time (sample to results) of under 48 h. An additional ‘pop-up’ testing centre was also implemented after seven weeks to improve access to testing and was located at a site around four kilometres from main campus, closer to student accommodation, with set up similar to the main testing centre.

### Registration

To facilitate transfer of results between laboratories and onwards to the national COVID-19 surveillance system and participants, the IT system within the local National Health Service (NHS) hospital was used. All participants were registered with a local primary care practice, on either a temporary or permanent basis, introducing a lead time of around seven days for registration before participants could request an appointment for testing. The practice generated requests for the hospital pathology system in order to process participants’ samples upon the booking of an appointment at the testing centre, and corresponding labels for samples were ready for participants to collect upon their arrival at the testing centre. Health data was kept within the NHS, and the practice informed participants of their results via SMS. No personal data relating to test results was held by the university.

### Booking system

The booking system utilised local IT systems which were already in place, using Microsoft 365. Appointment slots were opened up to a week in advance, but no sooner than 48 h in advance, allowing time for the primary care practice to process the pathology request and for printing of the corresponding pathology request. Data relating to booking of appointment slots was held within the University booking system.

### Confirmatory RT-PCR test in hospital for positive samples

**Fig. 2:**
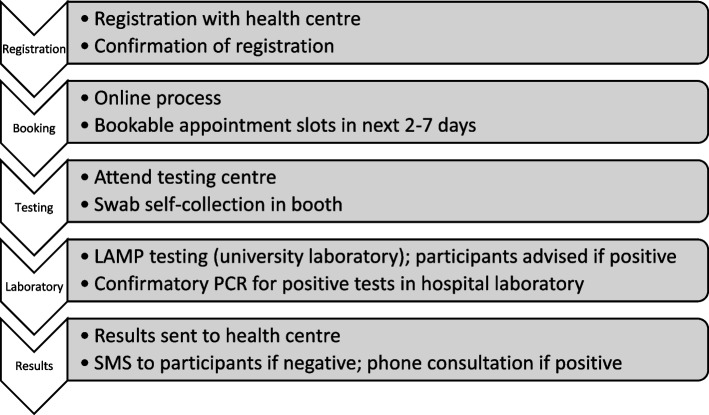
Steps in the testing process

To prevent false positive test results resulting in unnecessary isolation by participants, any tests returning as positive after the RT-LAMP test were transferred to the main hospital pathology laboratories for a confirmatory RT-PCR test. Participants were informed immediately of a positive RT-LAMP test by the university medical centre staff and advised to isolate pending the confirmatory test to prevent any delay in isolation. If the RT-PCR test was also positive, the primary care practice was informed at the earliest opportunity. They then informed the participant by telephone and provided appropriate public health advice, as well as giving advice on when to seek further medical advice. This result was also entered into the national Test and Trace system to allow contact tracing follow-up. Negative tests were communicated to primary care after RT-LAMP testing and results were then communicated to participants via SMS with public health advice. The steps involved in the process are detailed in Fig. 2.

### Time period of data collection

Appointments were held between 28 September and 18 December. These were initially daily, but moved to twice-weekly from 30 November due to decreased bookings, likely due to students leaving campus prior to the Christmas break and the concurrent national lateral flow testing introduced for students to travel safely.

### Data analysis

Data included for analysis in this paper are testing uptake, indicated by time period (weekly) and by location of swab collection, and the number of repeat bookings by individual participants. Quality assurance data are reported, namely the time of test to result and the internal control measure of total RNA. Positivity rate is also reported and refers to the proportion of tests returning a positive result. This can be a proxy measure of the infection rate in the population but does not provide the true infection rate.

## Results

### Testing uptake and positivity rate

Testing was available to all staff and students attending campus. A total of 1673 tests were performed during the study period. The total number of staff and students eligible for testing was initially around 10,000 people, although numbers attending campus decreased over the testing period due to implementation of national COVID-19 restrictions and students returning home as learning continued to be delivered remotely.

Data included the number of positive tests and cumulative number of tests, allowing calculation of a positivity rate. An additional testing location closer to student accommodation was operational from Week 8. Weekly data can be seen in Table 1.

**Table 1:** Number of tests per week by location and number of positive tests per week

Week number (week commencing date)	Number of tests (campus)	Number of tests (accommodation)	Total number of tests (weekly)	Total number of tests (cumulative)	Total number of positive test results (weekly)†	Total number of positive tests (cumulative)†
1 (28 Sept)	14	Site not operational	14	14	0	0
2 (5 Oct)	300	Site not operational	300	314	4	4
3 (12 Oct)	283	Site not operational	283	597	1	5
4 (19 Oct)	152	Site not operational	152	749	2	7
5 (26 Oct)	144	Site not operational	144	893	1	8
6 (2 Nov)	158	Site not operational	158	1051	0	8
7 (9 Nov)	72	Site not operational	72	1123	0	8
8 (16 Nov)	87	14	101	1224	0	8
9 (23 Nov)	100	23	123	1347	1	9
10 (30 Nov)	97	Site not operational	97	1444	0	9
11 (30 Nov)	95	Site not operational	95	1539	0	9
12 (14 Dec)	134	Site not operational	134	1673	0	9

^†^ all samples testing positive on RT-LAMP also tested positive using RT-PCR

During the period under investigation, there were 9 positive tests from a total of 1,673 tests. This gave an overall positivity rate of 0·54%. This is equivalent to a rate in the tested population of 538 cases per 100,000 over the duration of testing. The highest number of positive tests were returned at the start of this period, with four positive tests in the second week of testing (in early October). The time from test to result was usually between one and two working days depending on the requirement for confirmatory testing.

### False positive tests

Of the 1,673 tests conducted, nine tests were positive on RT-LAMP testing. These were all found to be positive on RT-PCR testing in the hospital laboratory, giving a false positive rate of 0%. Using the internal quality control of total RNA resulted in one sample being reported negative for total RNA (and SARS-CoV-2) and so a re-swab was requested, which was also negative.

### Repeat bookings

Testing was available for staff and students of the university as often as they requested it. Data for repeat bookings were generated by the number of bookings made by a specific email address. This is shown in Table 2. It can be seen that the majority of people booking a test (56%) used a unique email address to book one test. In a small minority of cases (5%), the same email address was used for more than four bookings.

**Table 2:** Number of repeat bookings made, by individual email address

Number of bookings	Proportion of unique email addresses (%)
1	56%
2	24%
3	10%
4	5%
4 +	5%

## Discussion

### Main findings of the study

The results indicate that the level of asymptomatic COVID-19 infection within the university population during the autumn term was equivalent to 538 cases per 100,000 of those tested. For comparison, national coronavirus data on the number of infections in the local authority are available [26], though this is from symptomatic testing and calculated on a weekly basis, making direct comparisons difficult. Between 23 October and 10 December, the number of incident cases of COVID-19 infection ranged from 217 to 525 per 100,000 in Leicester City. However, this showed a different pattern to the positive cases found in the university testing programme: infection rates in Leicester City increased from 23 October and peaked on 19 November, whereas the university testing programme showed a peak in the initial testing period, likely due to the convergence of students from many areas of the country onto one site, and was also observed at two other UK universities [27].

The absence of false positive tests from this programme demonstrates the value and usability of RT-LAMP as a molecular diagnostic tool for the detection of SARS-CoV-2 in an asymptomatic population. The internal control (total RNA) used to mitigate the number of false negatives, with only one individual requiring a repeat sample, showed that the method of sample extraction was also a feasible method of testing. These findings indicate that the accuracy of RT-LAMP can help to prevent harms from misdiagnosis or uncertain results.

This study describes two novel aspects of mass testing for asymptomatic COVID-19 infection: it shows that it is possible to rapidly set-up and implement a university testing programme for asymptomatic staff and students, and it shows that RT-LAMP testing gives comparable results to RT-PCR when examining those who test positive on RT-LAMP, with RT-LAMP providing results faster than RT-PCR testing. If it is decided to move towards a model whereby COVID-19 is viewed as an endemic, rather than pandemic, infection, it is likely that mass testing will remain a key tool in the armoury of measures to prevent widespread infection and disruption. This paper shows policymakers that a model of asymptomatic testing is feasible and palatable for staff and students on a UK university campus.

### Strengths and weaknesses of the study

A priority of the programme was that it should facilitate contact tracing and reduce onward transmission of COVID-19. Having a confirmatory RT-PCR test enabled positive samples to enter the national surveillance system and acted as a safety net if participants chose not to inform the university of the positive test. If participants informed the university of a positive test, support was available in the form of welfare checks, support with food and laundry, and a helpline for those who needed it.

Using an existing booking system rather than a bespoke system restricted the data that could be collected. As a result, there is no linked demographic data to describe the characteristics of those tested such as the proportions of staff and students receiving tests, the age and ethnicity of participants, and their location. This limits the generalisability of the results. Similarly, a significant limitation of the study was that there was no concurrent behavioural insight research, or research into the acceptability of the testing processes and procedures. This may have provided reasons for the decline in testing as well as testing behaviours.

The testing on campus was carried out alongside a rapidly changing national landscape with regards to testing, contact tracing and intense debate over the role of universities in the increasing rates of COVID-19 infection. A new policy relating to university students being offered two lateral flow tests prior to their leaving campus at the end of term may have impacted on the uptake of the university’s own tests. An additional aspect of the programme that may have affected uptake was the requirement to register with the local university health practice in order to be tested. Although registration was available on a temporary basis, and solely for the test, it is possible that this deterred some as they erroneously believed that this meant they could not continue to receive care from their usual family doctor.

### Strengths and weaknesses in relation to other studies

Asymptomatic testing was conducted in Liverpool from 6 November to 9 December 2020 in a pilot of community testing. The interim evaluation report [28] revealed that in lateral flow testing, a positivity rate of 0.73% was found among the asymptomatic population. This is higher than the rate of 0.54% found in this study, though the population tested in Liverpool included a wider range of age groups, and the pilot used lateral flow testing rather than RT-LAMP, which have different sensitivity rates.

From the trends in the bookings over time, it was clear that after an early peak in demand, this steadily dropped over the course of the university term. This was seen to an extent in a feasibility pilot conducted at the University of East Anglia which saw high initial drop-out [29]. This may show a fatigue effect or lack of engagement, but may also reflect the trends in student occupancy falling in university accommodation due to students returning home as most teaching remained online. This level of occupancy was not captured by the study, giving some uncertainty over the denominator population.

### Future research

Further evaluation of the testing programme could utilise the criteria used for national screening programmes [30]. Although the programme failed to meet several criteria to classify as a screening programme, such as the RT-LAMP test not yet being validated for SARS-CoV-2 and no randomised control trials, the test appeared to be acceptable to a proportion which may strengthen the case for minimally invasive tests being used for screening programmes as far as possible to increase uptake. A nuance of the university testing programme is that it focuses on the health of the wider population rather than individuals, in contrast to most recognised screening programmes.

Over half of the registrations for a COVID-19 test through the university testing system came from an email address which only registered for one test. Potential reasons for this include the test having a novelty value or being unacceptable to the population, or a belief that a negative test negated the need for further testing. There was a small number of individuals who registered for over four tests. Further research would be needed to examine the potential reasons for this.

Finally, a key limitation of this study is that it is not possible to say whether the asymptomatic testing programme contributed to limiting the rate of COVID-19 transmission on campus. This study was not designed to evaluate the frequency of testing or coverage of the target population required from a testing programme to effectively reduce transmission. Additional data such as individual case and contact follow-up would be required in order to ascertain whether the testing resulted in chains of transmission being broken. The testing programme is a small part of wider disease control with many complex elements, and so attempting to single out the effectiveness of one aspect of this would be open to many sources of confounding.

## Conclusions

This paper is the first to report prevalence of asymptomatic COVID-19 infection within a UK university population using RT-LAMP as a molecular diagnostic tool. With the publication of data from other universities, a richer picture will develop of the true extent of COVID-19 infection within university populations. It provides a comparison with the reported epidemiological data from the local community, which is rarely reported in other papers discussing COVID-19 infection in university populations.

This paper shows that it is possible to rapidly set up a universal university testing programme for COVID-19 in collaboration with local healthcare providers, and that RT-LAMP is an acceptable diagnostic tool. It details some of the key aspects of setting up such a programme and outlines the strengths and limitations of the programme implemented, providing lessons learned for others who wish to implement a similar testing programme for COVID-19 or other infectious diseases. Combined with existing evidence, our paper shows that comprehensive testing programmes can feasibly include large groups of the community who may not access standard testing services, and make use of novel technology.

## Supplementary Information

**Additional file 1:** **Figure 1.** Layout of testing centre, detailing testing booths and placement of testing equipment. **Figure 2.** Photo of individual testing booth and setup of equipment required. 

## Data Availability

The datasets used and analysed during the current study are available from the corresponding author on reasonable request.

## Abbreviations

MERS: Middle East respiratory syndrome
NHS: National Health Service
RT-LAMP: Reverse transcriptase loop-mediated isothermal amplification
RT-PCR: Reverse transcriptase polymerase chain reaction
SAGE: Scientific Advisory Group of Experts
SARS: Severe acute respiratory syndrome
