# Supplementary material for: How to design and implement a university-based COVID-19 testing programme? An evaluation of a novel RT-LAMP COVID-19 testing programme in a UK university
Source: BMC Health Serv Res. 2022 Dec 9;22:1502. doi: 10.1186/s12913-022-08717-5 (PMC9733160; doi:10.1186/s12913-022-08717-5)
Supplement: Supplementary file 1 — Additional file 1: Figure 1. Layout of testing centre, detailing testing booths and placement of testing equipment. Figure 2. Photo of individual testing booth and setup of equipment required. [file 12913_2022_8717_MOESM1_ESM.docx]

Supplementary Materials


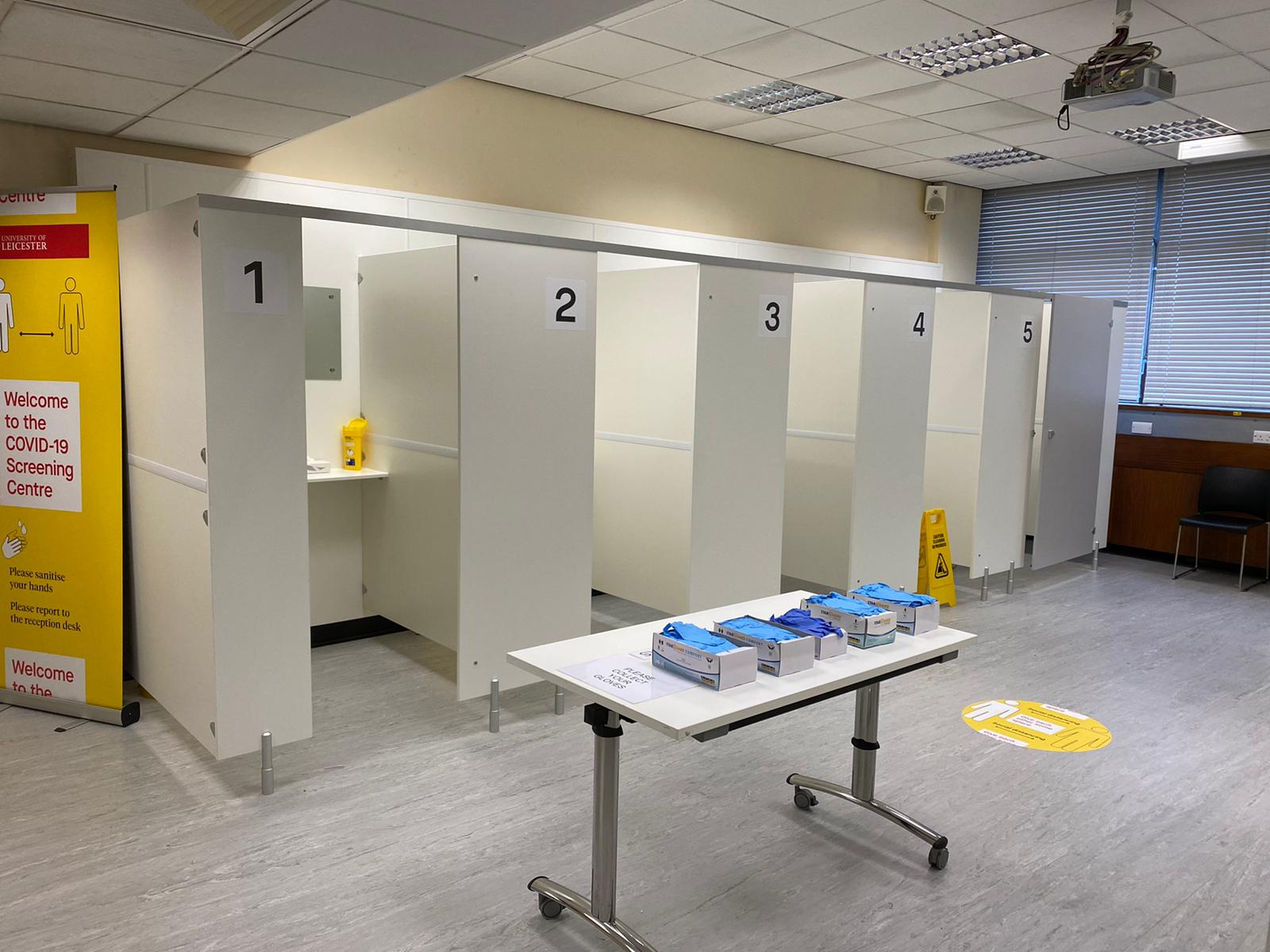


Figure 1: Layout of testing centre, detailing testing booths and placement of testing equipment


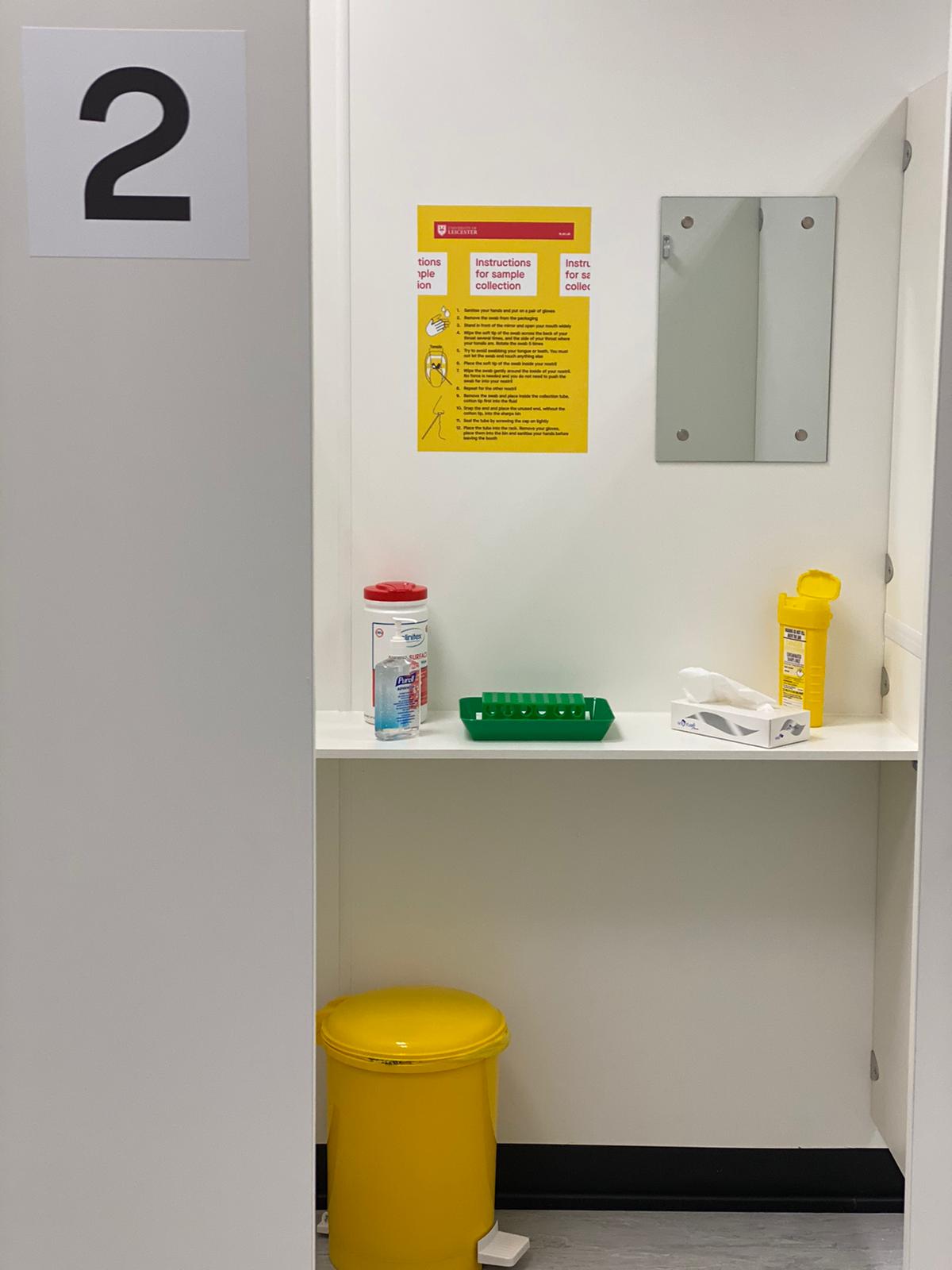


Figure 2: Photo of individual testing booth and setup of equipment required
